# Extended Spectrum *β*-Lactamase-Mediated Resistance and Antibiogram of *Pseudomonas aeruginosa* Isolates from Patients Attending Two Public Hospitals in Khartoum, Sudan

**DOI:** 10.1155/2020/2313504

**Published:** 2020-10-23

**Authors:** Thweaba Hassan Salih Omer, Samia Ali Mohammed Mustafa, Sagad Omer Obeid Mohamed

**Affiliations:** Faculty of Medicine, University of Khartoum, Alqasr Avenue P.O. Box 102, Khartoum, Sudan

## Abstract

Treating infections caused by *Pseudomonas aeruginosa* is challenging. In addition to its intrinsic ability to develop resistance to multiple classes of antibiotics, it also produces extended-spectrum *β*-lactamase (ESBL). Continuous update of the antibiograms is required to cope with the rate of the emergence of antibiotic resistance. This study aimed to determine the antimicrobial susceptibility pattern and to determine the frequency of ESBL production among the *P. aeruginosa* isolates from patients at two public military hospitals in Khartoum, Sudan. A total of 34 isolates of *P. aeruginosa* obtained from patients with diabetic septic foot wounds were tested for their antibiotic sensitivity patterns. Resistance occurred most commonly to ceftazidime (35%), followed by ciprofloxacin (20.6%) and piperacillin (14.7%). We found that 17.6% of the *P. aeruginosa* isolates were ESBL producers, but all of these isolates were sensitive to meropenem. The chi-squared test showed a significant association between the ESBL production and antimicrobial resistance to amikacin, ceftazidime, and piperacillin. Our findings strengthen previous reports in which aminoglycosides (amikacin) and carbapenems (meropenem) were found to be highly effective against *P. aeruginosa*. Our findings highlight the need for effective surveillance and antibiogram-guided antibiotic prescription.

## 1. Introduction

*Pseudomonas aeruginosa* is a clinically significant opportunistic Gram-negative, rod-shaped bacterium that is frequently implicated in community-acquired and nosocomial infections [1]. It has a natural resistance to several classes of antimicrobial agents, along with the ability to acquire resistance to all other treatment choices [2]. Several studies have reported that *P. aeruginosa* is becoming a multidrug-resistant microorganism [3, 4]. Diabetic patients are highly vulnerable to septic foot infection (DFI), which is a noteworthy source of morbidity, hospitalization, and lower limb amputation [5]. Among these patients, *P. aeruginosa* is a frequently isolated microorganism [6]. Staphylococcus aureus is the most common pathogen, and prevalence of methicillin-resistant S. aureus (MRSA) in DFIs is 15–30%. Other bacterial genera commonly found in DFIs include Streptococci, Enterococci, and Enterobacteriaceae [7, 8].

Extended-spectrum beta-lactamase (ESBL) confers a powerful loss of susceptibility to most of the beta-lactam antibiotics, including penicillin, cephalosporins, and monobactam [9]. Infections with ESBL-producing bacteria have been associated with poor outcomes [9]. Antimicrobial resistance threatens the effective prevention and treatment of an ever-increasing range of infections caused by the microorganisms and increases the hospital stay and thus leading to increasing economic burden [8]. Currently, the problem of resistance to beta-lactamase drugs and extended-spectrum cephalosporins is becoming a serious problem, and we envisage that shortly, the world will need new antibiotics to replace the existing ones [10].

The World Health Organization (WHO) and the Centers for Disease Control and Prevention (CDC) designated multidrug-resistant *P. aeruginosa* as a serious global threat and a priority for the research and development of new antibiotics [11–13]. Antibiograms aid in selecting empiric antibiotic therapy and in monitoring resistance trends over time [14].

Therefore, continuous updating and monitoring of the current patterns of the *P. aeruginosa* antibiotic sensitivity is required to cope with the rate of the emergence of antibiotic resistance. This study aimed to assess the current antibiotic sensitivity patterns of *P. aeruginosa* and to determine the frequency of ESBL producers among the *P. aeruginosa* isolates from patients with diabetic wound infections.

## 2. Materials and Methods

### 2.1. Study Design and Setting

A hospital-based cross-sectional study was conducted in September 2016 in Omdurman Military Hospital and Bahri Military Hospital in Khartoum State, Sudan. These settings were chosen because they are public tertiary healthcare facilities providing specialized clinical inpatient and outpatient services for a significant number of the Khartoum State population. The patients diagnosed with diabetes mellitus and presented to these hospitals with diabetic septic foot wounds were tested for the presence of *P. aeruginosa*. The wounds were swabbed via sterile swabs in the deepest part of the ulcers after the patients had undergone sharp debridement of all the necrotic and stained tissue until clean granulation tissue was obtained. The recovered isolates were submitted to the National Laboratory for Public Health in Khartoum for culture and sensitivity testing.

### 2.2. Isolation and Identification of *P. aeruginosa* Isolates

The clinical specimens were cultured on MacConkey agar, and those that were nonlactose fermenters were subcultured in Cetrimide agar. *P. aeruginosa* isolates were identified with reference to the colony morphology, production of pyocyanin pigment, negative Gram staining, catalase test, and oxidase test. The isolates of *P. aeruginosa* were evaluated for ESBL production by using the phenotypic confirmatory test. The Clinical and Laboratory Standards Institute (CLSI) guidelines for performing an ESBL confirmatory test involve testing cefotaxime and ceftazidime alone and in combination with clavulanate, which inhibits the activity of the ESBL enzyme and makes the organisms appear more sensitive with the drug + clavulanate combination [15]. An increase of ≥5 mm in the zone of inhibition for ceftazidime + clavulanic acid compared to ceftazidime alone was used to conﬁrm the presence of ESBL producers.

### 2.3. Antimicrobial Susceptibility Testing

We used the modified Kirby–Bauer disc diffusion technique on Mueller–Hinton agar (HiMedia, India) to perform antimicrobial sensitivity testing of the *P. aeruginosa* isolates according to the CLSI guidelines, 2015 [13] (Supplementary file 1). We assessed the antimicrobial susceptibility of *P. aeruginosa* to the commonly used antibiotics for treatment of *P. aeruginosa* in our setting (ceftazidime (30 *μ*g), ciprofloxacin (5 *μ*g), meropenem (10 *μ*g), piperacillin (100 *μ*g), amikacin (30 *μ*g), and cefepime (30 *μ*g)). All of these antibiotics were obtained from Bioanalyse Laboratories, Ankara, Turkey. Inhibition zones were measured and classified according to CLSI guidelines into resistant, sensitive, and intermediate.

### 2.4. Data Analysis

We used SPSS software version 21 (SPSS Inc., Chicago, IL, USA) for data analysis. Descriptive statistics of SPSS provided frequency tables and distribution of the variables. A chi-square test was conducted to determine the relationship between ESBL production and antimicrobial resistance. The *p* value of 0.05 was set as the significance level for all analyses.

### 2.5. Results

Out of 43 specimens, a total of 34 isolates of *P. aeruginosa* obtained from patients with diabetic septic foot wounds were analyzed in this study. Antimicrobial resistance occurred most commonly against ceftazidime (35.3%), and the resistance rates to ciprofloxacin, piperacillin, and meropenem were 14.6%, 20.6%, and 11.8%, respectively. Amikacin and meropenem displayed the lowest frequency of resistance (97% and 82.4% of the isolates were sensitive, respectively). Antimicrobial resistance profiles of the *P. aeruginosa* isolates are shown in Table 1.

Regarding ESBL production, the positive ESBL strains among the *P. aeruginosa* isolates were 17.6%. A significant association between the ESBL production and antimicrobial resistance pattern was detected (Table 2). The positive ESBL *P. aeruginosa* producers were more resistant to amikacin (*p*=0.028), ceftazidime (*p* ≤ 0.001), and piperacillin (*p*=0.016). However, all of the positive ESBL *P. aeruginosa* producers were sensitive to meropenem (Table 2).

## 3. Discussion

The development of antimicrobial resistance to commonly used antibiotics has been accelerated by the overuse of antibiotics worldwide [16]. Antibiograms are often used by clinicians to assess local susceptibility rates, as an aid in selecting empiric antibiotic therapy and in monitoring resistance trends over time within an institution. Updated knowledge of antimicrobial susceptibility profiles of clinical isolates could assist in designing the most appropriate treatment schedule against diabetic wounds infected with *P. aeruginosa* and help in curbing the expanding menace of antibiotic resistance. The effective treatment of patients with DFIs infected by *P. aeruginosa* depends on the administration of appropriate antibacterial agents [17]. The current study showed variable levels of antibiotic resistance for common classes of antibiotics used for treatment of *P. aeruginosa* infection in patients with DFIs.

It has been reported that the recommended drugs against *P. aeruginosa* are ciprofloxacin, antipseudomonal penicillins (ticarcillin and piperacillin), cephalosporins (ceftazidime and cefepime), aminoglycosides (amikacin and gentamicin), and carbapenems (imipenem and meropenem) [17]. Our findings strengthen conclusion from previous reports in that amikacin and meropenem are highly effective against *P. aeruginosa* [17–20]. However, we found that only near-half of the *P. aeruginosa* isolates were sensitive to piperacillin. Also, we found that 67.6% of the *P. aeruginosa* isolates was sensitive to ciprofloxacin.

A previous large prospective study by Wahab et al. in 2014 conducted over 18 months included all diabetic patients with infected wounds seen in a specialized center for diabetic patients in Khartoum found that 8.3% of them were infected by *P. aeruginosa*. Also, they concluded that DFIs with pseudomonas carry a higher risk for toe or lower limb amputation [17]. The antibiogram results of our study are consistent with the findings of Wahab et al., the aminoglycosides were found to be highly effective against *P. aeruginosa*, and ceftazidime was less effective against *P. aeruginosa* [20].

A previous study by Alsammani et al. in 2013 determined the antibiograms of some bacterial pathogens to ciprofloxacin and other commonly used antibiotics in Sudan. They showed that 75% of the *P. aeruginosa* isolates were susceptible to ciprofloxacin [21]. In this study, the antibiogram showed a resistance rate of 35.3% to ceftazidime, which is a lower rate than the one reported by Peshattiwar et al. [22]. In this study, meropenem retained good antipseudomonas activity, as reported by Aziz et al. previously [23]. Also, *P. aeruginosa* was most sensitive to amikacin with a resistance rate of only 2.9%.

The frequency of the ESBL among the isolates in this study is close to that reported by Peshattiwar et al. and Aziz et al. [22, 23]. All of the positive ESBL *P. aeruginosa* isolates were sensitive to meropenem. Similarly, it has been reported that carbapenems are the best antimicrobial agent for infections caused by ESBL producers [24–26]. The association between the antimicrobial resistance and the ESBL production could be attributed to the plasmids, which are responsible for ESBL production [24–26]. We found that all positive ESBL *P. aeruginosa* isolates were resistant to ceftazidime. This is explainable because ESBL producers are responsible for mediating resistance to *β*-lactams by hydrolyzing the oxyimino-*β*-cephalosporins [27]. ESBL is not always detectable in routine susceptibility tests, and failures to identify ESBL producers may delay the institution of suitable infection control measures and management [27].

We recommend routine surveillance on antibiotic resistance in hospitals and enhancing the practice of ESBL testing in conjunction with the antibiotic sensitivity. The results of this study suggest using carbapenems and aminoglycosides for treating *P. aeruginosa*. The main limitations of this study are the small numbers of isolates tested and the nature of a cross-sectional study done in one site. These considerations limit generalization for other settings in the country.

## 4. Conclusion

P. aeruginosa exhibited variable levels of resistance against different common classes of antibiotics. *P. aeruginosa* was mostly sensitive to aminoglycosides and carbapenems, which is consistent with the previous reports; resistance occurred most commonly to ceftazidime. Prevalence of ESBL-positive *P. aeruginosa* was 17.6%, and all of the positive ESBL isolates were sensitive to meropenem. The susceptibility data from this study should be considered when implementing empiric treatment strategies for diabetic wounds infected with *P. aeruginosa*.

## Figures and Tables

**Table 1:** Antibiotic susceptibilities of *P. aeruginosa* isolated at two hospitals in Khartoum.

Antibiotics		No.	Percent (%)
Amikacin	Sensitive	33	97.1
	Intermediate	0	0.0
	Resistant	1	2.9

Meropenem	Sensitive	28	82.4
	Intermediate	2	5.9
	Resistant	4	11.8

Ceftazidime	Sensitive	22	64.7
	Intermediate	0	0.0
	Resistant	12	35.3

Piperacillin	Sensitive	18	52.9
	Intermediate	11	32.4
	Resistant	5	14.7

Ciprofloxacin	Sensitive	23	67.6
	Intermediate	4	11.8
	Resistant	7	20.6

**Table 2:** Antibiotic susceptibilities of the positive ESBL *P*. *aeruginosa* isolates.

Antibiotics		ESBL production		*X* ^2^, *p* value
		Negative ESBL (*n* = 28) (%)	Positive ESBL (*n* = 6) (%)	
Amikacin	Sensitive	100.0	83.3	4.808, 0.028
	Resistant	0.0	16.7	

Meropenem	Sensitive	78.6	100.0	1.561, 0.458
	Intermediate	7.1	0.0	
	Resistant	14.3	0.0	

Ceftazidime	Sensitive	78.6	0.0	13.357, 0.001
	Resistant	21.4	100.0	

Piperacillin	Sensitive	64.3	0.0	8.228, 0.016
	Intermediate	25.0	66.7	
	Resistant	10.7	33.3	

Ciprofloxacin	Sensitive	75.0	33.3	4.478, 0.107
	Intermediate	10.7	16.7	
	Resistant	14.3	50.0	

## Data Availability

The datasets used during the current study are available from the corresponding author on reasonable request.

## Supplementary Materials

Supplementary Materials Figure 1: image for disk diffusion susceptibility test (DDS test).
