# Supplementary figures and images for: Extended Spectrum β-Lactamase-Mediated Resistance and Antibiogram of Pseudomonas aeruginosa Isolates from Patients Attending Two Public Hospitals in Khartoum, Sudan
Source: Int J Microbiol. 2020 Oct 23;2020:2313504. doi: 10.1155/2020/2313504 (PMC7607279; doi:10.1155/2020/2313504)

Figure 1: Image for Disk Diffusion Susceptibility test (DDS test
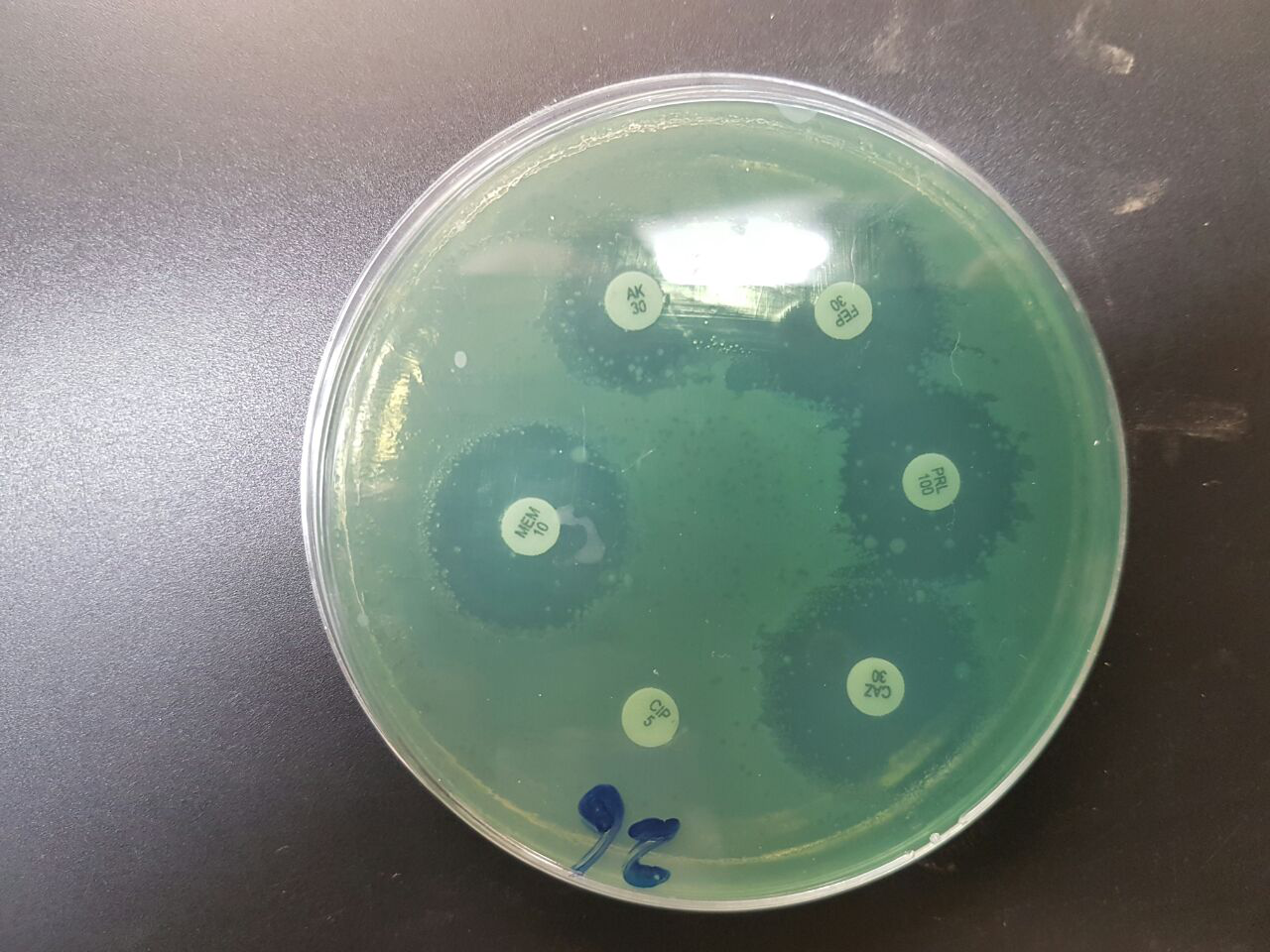
)

Supplement: Supplementary Materials — Figure 1: image for disk diffusion susceptibility test (DDS test). [file 2313504.f1.docx]
